# Typing of *Leishmania* isolates from vectors and leporids of the Madrid (Spain) outbreak

**DOI:** 10.1017/S0031182023001336

**Published:** 2024-02

**Authors:** Anna Fernández-Arévalo, Estela González, Cristina Ballart, Inés Martín-Martín, Silvia Tebar, Carme Muñoz, Maribel Jiménez, Ricardo Molina, Montserrat Gállego

**Affiliations:** 1 Secció de Parasitologia, Departament Biologia, Sanitat i Medi Ambient, Facultat Farmàcia i Ciències de l'Alimentació, Universitat de Barcelona, Barcelona, Spain; 2 Laboratorio de Entomología Médica, Centro Nacional de Microbiología, Instituto de Salud Carlos III, Majadahonda, Spain; 3 Institut de Salut Global de Barcelona (ISGlobal), Barcelona, Spain; 4 Servei de Microbiologia, Hospital de la Santa Creu i Sant Pau, Barcelona, Spain; 5 Institut de Recerca Biomèdica Sant Pau, Barcelona, Spain; 6 Departament de Genètica i Microbiologia, Universitat Autònoma de Barcelona, Bellaterra, Spain; 7 Centro de Investigación Biomédica en Red de Enfermedades Infecciosas (CIBERINFEC), Madrid, Spain

**Keywords:** *Leishmania*, outbreak, Spain, typing

## Abstract

In 2009, a large outbreak of leishmaniasis, associated with environmental changes, was declared near Madrid (Spain), in which *Phlebotomus perniciosus* was the vector, whereas the main reservoirs were hares and rabbits. Analysis of isolates from humans, vectors and leporids from the focus identified the *Leishmania infantum* ITS-Lombardi genotype. However, multilocus enzyme electrophoresis (MLEE), the reference technique for *Leishmania* typing, and sequencing of the *hsp70* gene, a commonly used marker, were not performed. In the present study, 19 isolates from *P. perniciosus* (*n* = 11), hares (*n* = 5) and rabbits (*n* = 3) from the outbreak area, all characterized as ITS-Lombardi in previous studies, were analysed by MLEE and *hsp70* sequencing. The *hsp70* results confirmed that all the analysed strains are *L. infantum*. However, by MLEE, 4 different zymodemes of *L. infantum* were identified based on variable mobilities of the NP1 enzyme: MON-34 (NP1^100^, *n* = 11), MON-80 (NP1^130^, *n* = 6), MON-24 (NP1^140^, *n* = 1) and MON-331 (NP1^150^, *n* = 1). The relative frequency of these zymodemes does not correspond to their usual occurrence in Spain. Moreover, MON-34 and MON-80 were found in *P. perniciosus*, hares and rabbits for the first time. These findings continue to provide insights into the outbreak and call for further studies with a higher number of strains.

## Graphical abstract

**Figure:**
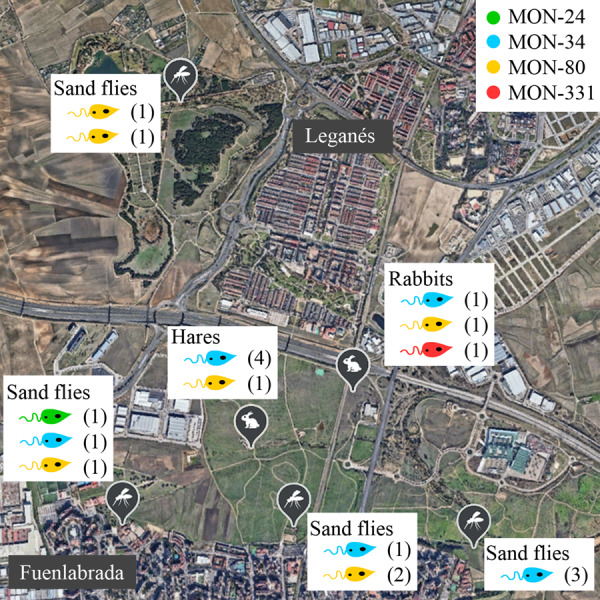

## Introduction

Leishmaniasis is a parasitological disease with worldwide distribution (World Health Organization, 2010). In the Mediterranean area, the disease is autochthonous, although imported cases are also found (Berriatua *et al*., 2021; Fernández-Arévalo *et al*., 2022; Van der Auwera *et al*., 2022). In Spain, the incidence reported for 2018 was 0.65 cases/100 000 habitants (Centro Nacional de Epidemiología, 2019), and in 2017 the estimated incidence rate was 0.86 for visceral leishmaniasis (VL), 1.04 for cutaneous leishmaniasis (CL) and 0.12 for mucocutaneous leishmaniasis, with an estimated underreporting of 14.7–20.2% for VL and 50.4–55.1% for CL (Humanes-Navarro *et al*., 2021). VL and CL are the main clinical manifestations in the country, but occasionally atypical CL or mucosal presentations are also diagnosed (Aliaga *et al*., 2003; Fernández Martínez *et al*., 2019). Although prevalence is higher in certain regions and seasons, the disease is present in most of Spain and cases are reported throughout the year (Fernández Martínez *et al*., 2019). The parasite responsible for autochthonous cases is *Leishmania infantum*, the principal domestic reservoir is the dog, and the main vectors are *Phlebotomus perniciosus* and *Phlebotomus ariasi* sand flies (Jiménez *et al*., 1995; Gállego *et al*., 2001; Martín-Sánchez *et al*., 2004; Berriatua *et al*., 2021). *Phlebotomus langeroni* has also been incriminated as a vector (Sáez *et al*., 2018) and different domestic and wild animals have been suspected or confirmed to be reservoirs (Azami-Conesa *et al*., 2021; Cardoso *et al*., 2021; Martín-Sánchez *et al*., 2021).

In 2009, the largest outbreak of leishmaniasis due to *L. infantum* ever detected in Europe was declared in Fuenlabrada, in the southwest Madrid region (Spain) (Arce *et al*., 2013). At its peak (2010–2014), more than 600 human cases were reported (Dirección General de Salud Pública – Consejería de Sanidad de la Comunidad de Madrid, 2015; Fernández Martínez *et al*., 2019) and asymptomatic carriers were also detected (Molina *et al*., 2020). The great majority of the patients were immunocompetent adults living in the area who presented cutaneous forms of the disease (Arce *et al*., 2013; Carrillo *et al*., 2013). However, children and immunocompromised individuals were also affected, and VL cases and rare clinical manifestations were observed, as well as a high susceptibility of the immigrant population born in Sub-Saharan Africa (Arce *et al*., 2013; Gomez-Barroso *et al*., 2015; Horrillo *et al*., 2015). Demographic and environmental changes, such as the construction of a vast green park connecting different urban zones, favoured the proliferation of *P. perniciosus*, hares (*Lepus granatensis*) and rabbits (*Oryctolagus cuniculus*), which were incriminated as reservoirs, and led to the outbreak (Molina *et al*., 2012; Carrillo *et al*., 2013; Jiménez *et al*., 2014; González *et al*., 2021).

Over the years, the leishmaniasis cases, reservoirs, vectors and parasites associated with the outbreak have been extensively investigated. However, few studies have characterized the parasites beyond species level. Two studies used the internal transcriber spacer (ITS) to type 3 and 4 strains isolated from hares and rabbits, respectively (Molina *et al*., 2012; Jiménez *et al*., 2014). All strains shared the ITS sequence of the strain MHOM/ES/87/Lombardi (AJ000295), this strain corresponding to zymodeme MON-24 of *L. infantum* (Chicharro *et al*., 2013). Similar results were obtained for 6 DNA samples and 67 isolates from sand flies when ITS2 was tested (Jiménez *et al*., 2013; González *et al*., 2017). In another study, 31 human isolates related to the outbreak were characterized by ITS and *haspb* (k26) markers (Chicharro *et al*., 2013). All strains were identified as ITS-Lombardi, but when combined with the *haspb* (k26) results, 4 different genotypes were detected, L-920 being the most prevalent. Given this background, the aim of the present study was to dig deeper into the characterization of non-human strains from the leishmaniasis outbreak using multilocus enzyme electrophoresis (MLEE), the technique most extensively used in epidemiological studies of leishmaniasis foci and the gold standard recommended by the WHO for this purpose (World Health Organization, 2010). Additionally, strains were also analysed by PCR-sequencing of the heat shock protein 70 (*hsp70*), one of the main markers used for *Leishmania* characterization (Fernández-Arévalo *et al*., 2022; Van der Auwera *et al*., 2022).

## Materials and methods

### Strains

Nineteen strains previously isolated from sand flies and leporids captured at different locations during the leishmaniasis outbreak area of Fuenlabrada (Spain) between 2011 and 2014 were selected for analysis. Eleven of them were isolated from 11 field-captured *P. perniciosus* in 2012 (*n* = 4), 2013 (*n* = 4) and 2014 (*n* = 3). The sand flies specimens had been captured in 4 collecting stations placed in institutional facilities: 3 located in the municipality of Fuenlabrada, in the border zone of the green park, (ATE-station *n* = 3, BOS-station *n* = 3, JIC-station *n* = 3) and 1 in the central area of the park in the municipality of Leganés (POL-station *n* = 2) (González *et al*., 2017). The other 8 strains were isolated from colony specimens of *P. perniciosus* used in direct xenodiagnosis of hares and rabbits. Five of them were isolated from 3 hares living in the park captured in 2011/12 and 3 were obtained from 3 rabbits captured in the border zone in 2013 (Molina *et al*., 2012; Jiménez *et al*., 2014). All the isolates had been previously characterized by ITS sequencing as the Lombardi type (Molina *et al*., 2012; Jiménez *et al*., 2013, 2014; González *et al*., 2017).

The strains were thawed and cultured in Schneider's insect medium supplemented with 20% fetal bovine serum and 1% sterile human urine until the exponential growth phase (Fernández-Arévalo *et al*., 2022).

### Hsp70 gene sequencing

DNA from the cultured strains was obtained by the QIAmp DNA Mini Kit (Qiagen) following the manufacturer's instructions. Amplification was done using previously described primers and cycling conditions (Van der Auwera *et al*., 2013; Fernández-Arévalo *et al*., 2022). PCR products were enzymatically purified using EXOSAP-IT (Affymetrix USB) and double-strand sequenced by the Sanger method at the Scientific and Technologic Centres of the University of Barcelona. The obtained sequences were edited using MEGA X software (Kumar *et al*., 2018).

### MLEE

Protein extracts were obtained from mass cultures as previously described (Piarroux *et al*., 1994). The strains were analysed by MLEE using a panel of 15 enzymes [malate dehydrogenase (MDH) E.C 1.1.1.37, malic enzyme (ME) EC 1.1.1.40, isocitrate dehydrogenase (ICD) EC 1.1.1.42, phosphogluconate dehydrogenase (PGD) EC 1.1.1.44, glucose-6-phosphate dehydrogenase (G6PD) EC 1.1.1.49, glutamate dehydrogenase (GLUD) EC 1.4.1.3, NADH diaphorase (DIA) EC 1.6.2.2, nucleoside phosphorylase purine (NP) − 1 and 2 − EC 2.4.2.1, glutamate oxaloacetate transaminase (GOT) − 1 and 2 − EC 2.6.1.1, phosphoglucomutase (PGM) EC 2.7.5.1, fumarate hydratase (FH) 4.2.1.2, mannose phosphate isomerase (MPI) EC 5.3.1.8 and glucose phosphate isomerase (GPI) EC 5.3.1.9] (Rioux *et al*., 1990). The samples were run on starch gel and revealed under suitable conditions for each enzyme. Migration distances were manually measured and compared with those of the marker strains (Supplementary Table S1) to assign the corresponding electromorphs and zymodemes. Once all enzyme mobilities were determined, the strains were tested again with a second batch of protein extract to confirm the results. The isoelectric focusing technique was also used to confirm the NP1 mobility (Piarroux *et al*., 1994).

## Results

Analysis of the *hsp70* gene showed that all the analysed strains shared the same genotype, presenting the main sequence reported for *L. infantum* strains (GenBank accession numbers OQ747159–OQ747177). Neither SNPs nor heterozygous positions were detected.

The strains produced different MLEE patterns. All of them shared an electrophoretic mobility (EM) of 104 for the MDH enzyme (MDH^104^) and an EM of 100 for the other enzymes except NP1 (EM: 100, 130, 140, 150). Thus, NP1 was the only enzyme associated with enzymatic polymorphisms in *Leishmania* strains from the outbreak. NP1^100^ was observed in 11 out of the 19 strains, which were therefore identified as *L. infantum* zymodeme MON-34. Six strains shared NP1^130^ (MON-80), whereas NP1^140^ was observed in 1 strain (MON-24), and NP1^150^ in the remaining strain (MON-331; also known as GR-19 and MON-24 var NP1^150^) (Table 1).

**Table 1.** Results of MLEE *Leishmania infantum* characterization related to host, location and year of isolation

Zymodeme	Original host	Location^a^	Year	WHO code
MON-24 (*n* = 1)	*Phlebotomus perniciosus*	JIC station	2013	IPER/ES/2013/LEMSJIC1FF4
MON-34 (*n* = 11)	*Phlebotomus perniciosus*	ATE station	2012	IPER/ES/2012/LEMATE1FL2
			2013	IPER/ES/2013/LEMATE1FL6
			2014	IPER/ES/2014/LEMATE1FL8
		BOS station	2012	IPER/ES/2012/LEMBOS1FL1
		JIC station	2012	IPER/ES/2012/LEMSJIC1FF1
		POL station	2013	IPER/ES/2013/LEMPOL2FL21
	*Oryctolagus cuniculus*	Border zone	2013	IPER/ES/2013/LEMC6
	*Lepus granatensis*	Park	2012	IPER/ES/2012/LEML34BX
				IPER/ES/2012/LEML34DX
				IPER/ES/2012/LEML40AX
				IPER/ES/2012/LEML40BX
MON-80 (*n* = 6)	*Phlebotomus perniciosus*	BOS station	2013	IPER/ES/2013/LEMBOS1FL10
			2014	IPER/ES/2014/LEMBOS1FL32
		JIC station	2014	IPER/ES/2014/LEMSJIC1FF19
		POL station	2012	IPER/ES/2012/LEMPOL2FL7
	*Oryctolagus cuniculus*	Border zone	2013	IPER/ES/2013/LEMC17
	*Lepus granatensis*	Park	2011	IPER/ES/2011/LEML1D18
MON-331 (*n* = 1)	*Oryctolagus cuniculus*	Border zone	2013	IPER/ES/2013/LEMC3

a ‘JIC’, ‘BOS’ and ‘ATE’ are the codes used to identify the collecting stations placed in the border zone of the park in the municipality of Fuenlabrada, and ‘POL’ the code the collecting station placed in the centre of the park in the municipality of Leganés.

**Figure 1.**
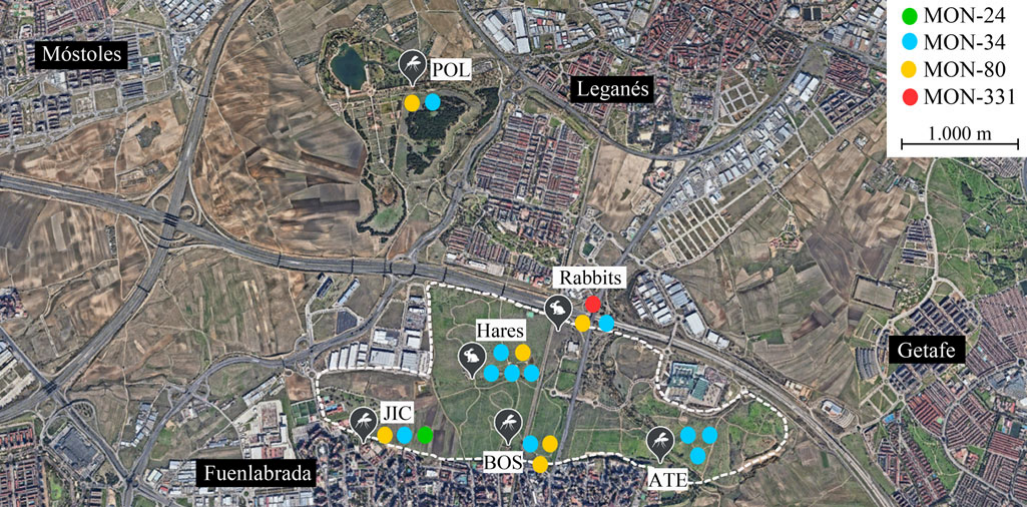
Distribution of *Leishmania infantum* zymodemes and hosts analysed in the leishmaniasis outbreak from Madrid region (Spain). Each circle represents 1 strain coloured according to its zymodeme. POL (placed in the municipality of Leganés), JIC, BOS and ATE (in the municipality of Fuenlabrada) are sand flies collecting stations where *L. infantum* strains were isolated from *P. perniciosus*. Rabbits were captured all around the perimeter of the Bosquesur park (marked with a dotted line) while hares were captured inside the park. The map image was taken from Google Earth (https://earth.google.com).

No correlations were apparent between the zymodeme and host, year or location (Table 1, Fig. 1). The only MON-24 strain was isolated from a *P. perniciosus* specimen in 2013. MON-34 and MON-80 zymodemes were found in rabbits, hares and sand flies from all collecting stations and in all years. Finally, the zymodeme MON-331 was detected in 1 rabbit. If the strains are grouped according to the collecting station and animal reservoir of origin, ‘JIC-station’, ‘POL-station’ and ‘rabbits’ present the most diversity, as each strain in these groups belonged to a different zymodeme. In contrast, all ‘ATE-station’ strains were MON-34 (Fig. 1).

## Discussion

In an unexpected outbreak in an endemic area, identification of the aetiological agent gains importance. Human leishmaniasis can be caused by about 20 of the 56 *Leishmania* species described (Akhoundi *et al*., 2016) and in the current context of globalization and climate change, the presence of imported species cannot be ruled out. In the leishmaniasis outbreak in Fuenlabrada, although part of the immigrant population was highly affected (Arce *et al*., 2013; Gomez-Barroso *et al*., 2015), the causative species was promptly identified as the autochthonous *L. infantum* (Chicharro *et al*., 2013).

When performing characterization studies within the *L. donovani* complex, and specifically for *L. infantum* strains, one of the major obstacles is the low intraspecific diversity (Maurício, 2018; Fernández-Arévalo *et al*., 2020). Different techniques have been used for the intraspecific analysis of *L. infantum*, including MLEE, multilocus microsatellite typing, multilocus sequence typing (MLST), randomly amplified polymorphic DNA, PCR-RFLP, and PCR-sequencing (Toledo *et al*., 2002; Ferroglio *et al*., 2006; Montoya *et al*., 2007; Aït-Oudhia *et al*., 2011; Van der Auwera and Dujardin, 2015). Depending on the technique and genetic markers used, a different resolution of genetic diversity is achieved. For instance, microsatellite typing provides extremely variable results, whereas single gene PCR-sequencing or PCR-RFLP techniques often show limited diversity (Van der Auwera and Dujardin, 2015). Accordingly, the 19 studied strains from the outbreak were genetically identical by both *hsp70* and ITS sequencing. The *hsp70* marker is very reliable for *Leishmania* species identification, but its resolution is too low to perform intraspecies characterization within the *Leishmania donovani* complex (Fernández-Arévalo *et al*., 2020). The ITS region can differentiate strains below the species level, and it has been correlated with the geographical origin. Evidence for this is the distinction of the Lombardi genotype from the ITS-A genotype, which is also common in the area. However, in *L. infantum* or *L. donovani* complex strains, ITS shows a lower degree of polymorphism than in other complexes, not being sufficiently resolving for outbreak studies (Kuhls *et al*., 2005; Fernández-Arévalo *et al*., 2022). Sequencing results can be improved in terms of resolution and robustness by a MLST approach (Kuhls and Mauricio, 2019). However, the lack of such studies in the area, together with the absence of a consensus scheme providing sufficient resolution for *L. infantum sensu stricto* strains from the same geographical origin, hinders, in this case, an integrative understanding of the results. As a side note, it may be interesting to mention that a recent study based on complete maxicircle coding regions, which could be considered kind of MLST, detected 2 distinct *L. infantum* populations among humans in the Madrid region, in almost perfect agreement with ITS typing (Solana *et al*., 2022).

MLEE offers an intermediate and manageable level of discrimination for these species (Fernández-Arévalo *et al*., 2022) and it has been extensively used in epidemiological studies to assess the geographical distribution of isolates and clinical presentations, and to elucidate vectors, and reservoirs (Pratlong *et al*., 2004; Montoya *et al*., 2007; Aït-Oudhia *et al*., 2011; Haouas *et al*., 2012). However, nowadays, this methodology is falling out of use. The requirement of mass culture of the strains and its manual and laborious protocol make it a costly and time-consuming technique, limiting its use to specific situations. MLEE is not the most suitable technique for inferring taxonomy, as it relies on phenotypic traits that may not be consistent with genetics. But, when applied for *L. infantum* intraspecific typing, MLEE can provide valuable information on reservoirs and vectors, allowing comparison between foci, and providing data to fill epidemiological gaps. Here lies the interest in using MLEE to characterize the strains isolated from the vectors and reservoirs implicated in the leishmaniasis outbreak in Madrid.

The use of MLEE to type *L. infantum* has shown that strains zymodemes in humans may belong to viscerotropic, dermotropic or both (Pratlong *et al*., 2004). In the canine reservoir, zymodeme MON-1 is predominant and few others have been isolated, giving rise to the suspicion of anthroponotic transmission cycles in some foci (Aït-Oudhia *et al*., 2011). MON-1 is also the main zymodeme identified in isolates from other reservoir hosts, such as cats, foxes, rats, horses and even rabbits (Gállego *et al*., 2001; Martín-Sánchez *et al*., 2004; Campino *et al*., 2006; Díaz-Sáez *et al*., 2014). In contrast, greater variation has been observed in strains isolated from sand flies (Gállego *et al*., 2001; Martín-Sánchez *et al*., 2004). In the strains included in the present study, MLEE analysis revealed 4 different zymodemes (MON-24, MON-34, MON-80 and MON-331), which vary from each other by a single enzyme (NP1). Except for MON-331, these zymodemes are commonly found in the Madrid region, as well as in the rest of Spain and the Mediterranean basin (Jiménez *et al*., 1995; Chicharro *et al*., 2003, 2013; Pratlong *et al*., 2013). Nevertheless, the frequency of the zymodemes detected here differs from what is usually found in Spain. None of the strains analysed belonged to MON-1, the predominant *L. infantum* zymodeme, which may be found in ITS-Lombardi strains. Moreover, only a single strain was identified as MON-24, the second most frequent human *L. infantum* zymodeme including in the area of the outbreak (Jiménez *et al*., 1995; Chicharro *et al*., 2003, 2013), where it was isolated from a *P. perniciosus*. Other studies carried out in the Mediterranean basin have identified MON-24 in strains from cases of human VL and CL and canine leishmaniasis, as well as from sand flies (Gállego *et al*., 2001; Martín-Sánchez *et al*., 2004; Campino *et al*., 2006; Haouas *et al*., 2012). Surprisingly, the most prevalent zymodeme in our study was MON-34 (11/19 strains), followed by MON-80 (6/19 strains), both detected in hares, rabbits and sand flies captured at all the collection points. According to the literature, zymodeme MON-34 has been isolated in human CL and VL cases and in animals (dogs and racoons), but it has not been reported in sand flies until now (Jiménez *et al*., 1995; Harrat *et al*., 1996; Gállego *et al*., 2001; Martín-Sánchez *et al*., 2004; Montoya *et al*., 2007; Pratlong *et al*., 2013; Fernández-Arévalo *et al*., 2022). MON-80 is less frequent in Spain, and it has been isolated mainly in humans with VL and CL throughout its distribution range (Gállego *et al*., 2001; Chicharro *et al*., 2003; Martín-Sánchez *et al*., 2004; Campino *et al*., 2006; Pratlong *et al*., 2013). To the best of our knowledge, only 1 study in North Africa has reported MON-80 in dogs (Benikhlef *et al*., 2009), and no previous reports describe it in sand flies, although it has been demonstrated experimentally that *P. perniciosus* could be a potential vector in the outbreak area (Remadi *et al*., 2018). Therefore, this is the first time that MON-34 and MON-80 strains have been isolated from *P. perniciosus* and leporids.

Regarding the zymodeme MON-331, found here in a rabbit, only 1 strain has been described before (GR-19, MON-24 var NP1^150^), in an HIV-positive human with VL (Martín-Sánchez *et al*., 2004). Although rare, NP1^150^ has been detected in other zymodemes of the *L. donovani* complex in isolates from Kenia, Sudan and China (Pratlong *et al*., 2013).

Unfortunately, no human isolates from this outbreak have been typed by MLEE. Only 1 study has characterized human strains from the Fuenlabrada outbreak (Chicharro *et al*., 2013), using ITS and *haspb* (k26) as molecular markers, also identifying 4 strain types (by *haspb* (k26)) that shared the ITS-Lombardi sequence. There is no apparent correlation between the isoenzymes and ITS sequences. Besides MON-34, MON-80 and MON-331, reported here, ITS-Lombardi has been described in MON-1, MON-24 and MON-27 strains (Chicharro *et al*., 2013; Fernández-Arévalo *et al*., 2022), whereas ITS sequence types other than Lombardi have been observed in MON-1, MON-24 and MON-34 strains (Kuhls *et al*., 2005; Chicharro *et al*., 2013; Fernández-Arévalo *et al*., 2022). As far as we know, there is no correlation between *haspb* (k26) and MLEE either. Based on the available data, it seems that human, reservoir and vector strains from the Fuenlabrada outbreak are similar, but it is not possible to ascertain any relationship between them.

According to MLEE background records, it is likely that the human strains from the outbreak could correspond to MON-34, MON-80 and MON-24. Regarding clinical manifestations, all these zymodemes have been detected in immunocompetent and immunocompromised individuals with VL and CL (Jiménez *et al*., 1995; Gállego *et al*., 2001; Chicharro *et al*., 2003; Martín-Sánchez *et al*., 2004; Pratlong *et al*., 2013), cases of which were also reported in the outbreak (Arce *et al*., 2013; Carrillo *et al*., 2013; Horrillo *et al*., 2015). However, despite providing data of unquestionable interest, it is unlikely that MLEE would be performed in human samples due to the performance drawbacks mentioned above.

When gathering all the available information about *L. infantum* in the Fuenlabrada outbreak, various questions arise. On one hand, the typing results reflect a certain homogeneity among the strains involved, although they belong to different zymodemes, and the strain types found are not new or exclusive to the area, which casts doubt on the emergence and transmission of a specific strain type as the detonator of the outbreak. On the other hand, the strains isolated from *P. perniciosus* in the area have shown higher virulence in the transmission of *L. infantum* to hamsters in *ex vivo* tests with experimentally infected *P. perniciosus* and in *in vivo* studies with the hamster and mouse model (Domínguez-Bernal *et al*., 2014; Martín-Martín *et al*., 2015; Mas *et al*., 2021). However, the strains do not seem to cause disease in leporids (Molina *et al*., 2012; Jiménez *et al*., 2014), nor were they found in local dogs, and some exhibit less virulent behaviour in canine cells (Mas *et al*., 2020). It remains uncertain if there was a selection of these strain types in the leporid reservoir or if they were simply the strain types present in the area at the onset of the outbreak, which arose from the interaction between large populations of vectors, reservoirs and humans. Furthermore, it is unknown if these strains are circulating among leporids in other endemic regions.

The lack of comparable characterization studies hampers a global understanding of the situation. To fully determine the epidemiological characteristics of the Fuenlabrada outbreak and the circulating strains in the region, more comprehensive studies with a higher number of isolates from humans, vectors and reservoirs from a range of *L. infantum* endemic regions are needed, as well as more robust and discriminative techniques.

## Supporting information

Fernández-Arévalo et al. supplementary material Fernández-Arévalo et al. supplementary material

## Data Availability

Sequences are available in GenBank under accession numbers OQ747159-OQ747177.

## Supplementary material

The supplementary material for this article can be found at https://doi.org/10.1017/S0031182023001336
