# Supplementary material for: Typing of Leishmania isolates from vectors and leporids of the Madrid (Spain) outbreak
Source: Parasitology. 2023 Dec 18;151(2):213–9. doi: 10.1017/S0031182023001336 (PMC10941034; doi:10.1017/S0031182023001336)
Supplement: Fernández-Arévalo et al. supplementary material [file S0031182023001336sup001.docx]

**Supplementary Table S1.** *Leishmania infantum* reference strains used as markers in the MLEE analysis together with their electromorphs for the different loci.

| **WHO code** | **Zymodeme** | **MDH** | **PGD** | **G6PD** | **GLUD** | **NP1** | **GOT1** | **GOT2** | **GPI** |
| --- | --- | --- | --- | --- | --- | --- | --- | --- | --- |
| MHOM/FR/78/LEM-75 | MON-1 | 100 | 100 | 100 | 100 | 100 | 100 | 100 | 100 |
| MHOM/FR/80/LEM-189 | MON-11 | 104 | 100 | 105 | 100 | 130 | 100 | 100 | 100 |
| MHOM/DZ/82/LIPA59 | MON-24 | 104 | 100 | 100 | 100 | 140 | 100 | 100 | 100 |
| MHOM/ES/90/BCN-61 | MON-28 | 104 | 100 | 102 | 100 | 140 | 100 | 100 | 100 |
| MHOM/ES/81/BCN1 | MON-29 | 104 | 100 | 105 | 100 | 140 | 100 | 100 | 100 |
| MHOM/DZ/83/LIPA120  MHOM/FR/84/LEM-538 | MON-34 | 104 | 100 | 100 | 100 | 100 | 100 | 100 | 100 |
| MHOM/DZ/83/LEM-425 | MON-80 | 104 | 100 | 100 | 100 | 130 | 100 | 100 | 100 |
| MHOM/SD/62/3S | MON-81 | 112 | 100 | 100 | 100 | 150 | 100 | 100 | 100 |
| *MHOM/IN/00/DEVI | MON- 2 | 104 | 93 | 100 | 100 | 140 | 113 | 113 | 100 |
| *MHOM/CN/00/WANGJIE-1 | MON-35 | 104 | 100 | 100 | 150 | 150 | 113 | 113 | 86 |

Electromorph values are relative to those of *L. infantum* zymodeme MON-1, which has an electrophoretic mobility (EM) of 100 for all 15 enzymic loci. For the strains in the table, the non-shown loci have an EM = 100. (*) *Leishmania donovani* strains.
